# Retrospective Proteomic Analysis of Cellular Immune Responses and Protective Correlates of p24 Vaccination in an HIV Elite Controller Using Antibody Arrays

**DOI:** 10.3390/microarrays5020014

**Published:** 2016-06-02

**Authors:** Suneth S. Perera, Bin Wang, Arturo Damian, Wayne Dyer, Li Zhou, Viviane Conceicao, Nitin K. Saksena

**Affiliations:** 1 Department of Medicine, University of Sydney, Sydney 2000, Australia; perera.suneth@gmail.com (S.S.P.); bin.wang1@unsw.edu.au (B.W.); li.zhou@health.nsw.gov.au (L.Z.); vickanc@hotmail.com (V.C.); 2 Department of Cytogenetics, Children’s Hospital at Westmead, Sydney 2000, Australia; Arturo.damian@health.nsw.gov.au; 3 Australian Red Cross Blood Service, 17 O’Riordan Street, Alexandria NSW 2015 and School of Medical Sciences, (Faculty of Medicine) University of Sydney, Sydney 2000, Australia; Wayne.dyer@sydney.edu.au

**Keywords:** Antibody microarray, HIV, immune responses, chemokines, cytokines, Acquired immune deficiency syndrome (AIDS), p24 vaccine, monocyte, CD4^+^ T, CD8^+^ T cells

## Abstract

Background: HIV p24 is an extracellular HIV antigen involved in viral replication. Falling p24 antibody responses are associated with clinical disease progression and their preservation with non-progressive disease. Stimulation of p24 antibody production by immunization to delay progression was the basis of discontinued p24 vaccine. We studied a therapy-naive HIV+ man from Sydney, Australia, infected in 1988. He received the HIV-p24-virus like particle (VLP) vaccine in 1993, and continues to show vigorous p24 antigen responses (>4% p24-specific CD4^+^ T cells), coupled with undetectable plasma viremia. We defined immune-protective correlates of p24 vaccination at the proteomic level through parallel retrospective analysis of cellular immune responses to p24 antigen in CD4^+^ and CD8^+^ T cells and CD14^+^ monocytes at viremic and aviremic phases using antibody-array. We found statistically significant coordinated up-regulation by all three cell-types with high fold-changes in fractalkine, ITAC, IGFBP-2, and MIP-1α in the aviremic phase. TECK and TRAIL-R4 were down-regulated in the viremic phase and up-regulated in the aviremic phase. The up-regulation of fractalkine in all three cell-types coincided with protective effect, whereas the dysfunction in anti-apoptotic chemokines with the loss of immune function. This study highlights the fact that induction of HIV-1-specific helper cells together with coordinated cellular immune response (*p* < 0.001) might be important in immunotherapeutic interventions and HIV vaccine development.

## 1. Introduction

HIV-1 p24 response is known to occur in HIV-infected individuals, but it wanes with time in concomitance with viremia [1,2]. In contrast, this response is, more intact in aviremic therapy-naïve HIV+ long-term non-progressors [1]. Taking this into account, it is believed that therapeutic immunization HIV-1 p24 antigen in HIV infection will induce HIV-specific immune responses, which will be durable and capable of controlling HIV disease progression. Moreover, it is known that cellular immune responses against HIV Gag capsid protein p24 are durable and robust and therefore important at various stages of HIV disease. Gag-specific T-cell responses associate with favorable clinical course [3,4,5,6]. However, the correlates of this protective immunity remain to be elucidated. As waning p24 antibody responses are associated with clinical disease progression [1], stimulating p24 antibody production with the use of therapeutic immunization may delay clinical progression. This was the basis of a p24 vaccination trial for HIV-infected individuals initiated in the early 1990s, but the vaccine trial was inconclusive [7]. Therapeutic vaccination has been proposed as a mechanism for stimulating the immune response in patients with chronic HIV infection [8]. Studies suggest that immunization with HIV antigens can delay CD4^+^ T cell decline and lower the rate of viral DNA increase in asymptomatic HIV-1 infected people [9]. However, other studies have failed to show any significant improvement in surrogate markers with the use of a therapeutic vaccination [10,11].

We recently found an HIV-infected, therapy-naïve non-progressor, who was enrolled in the HIV p24 VLP vaccine trial in 1993 in Australia. Patient still maintains CD4^+^ T cell counts (700/µL blood), CD8^+^ T cell counts (1400/µL blood), <20 copies of HIV RNA/mL plasma (January 2013) and harbors strong HIV gag-p24 specific proliferative responses, with >4% of antigen-specific cells [12,13] after 20 years of HIV-p24 vaccination, but it is unclear what his p24 responses were prior to vaccination in 1993. Here we describe cellular responses to p24 at the protein level in CD4^+^, CD8^+^ T cells and monocytes of this p24-vaccinated individual at the level of 274 cytokines, chemokines, their receptors, ligands, and associating proteins in order to define protective correlates of p24 vaccine. Throughout the course of HIV infection, the study individual has been therapy naïve with below detectable levels of HIV RNA in plasma until 2011. In a rare clinical event, in mid-2011, the study individual experienced a viremia blip showing 563 copies of HIV RNA/mL plasma, which naturally subsided fairly quickly to below detectable levels. We have analyzed p24 antigen responses at both viremic and aviremic phases of this individual collected in 2011. To our knowledge this study shows, for the first time, the protective immune correlates of HIV-p24 vaccination at the protein level in a rare therapy naive HIV+ non-progressor, who may have responded to the p24 vaccine. Contrary to these previous studies, which focused on a single arm of the immune response, this study simultaneously analyzed CD4^+^, CD8^+^ T cells, and CD14^+^ monocytes. Our data are unique in showing a detailed snapshot of the immune correlates functioning during viremic and aviremic phases of this p24-vaccinated individual with a clear distinction between the cell types governing each of these phases of HIV disease and the vital role of coordinated immune response of all three cell types in HIV disease and its control.

## 2. Methods and Materials

### 2.1. Clinical History of the Study Subject

**Figure 1:**
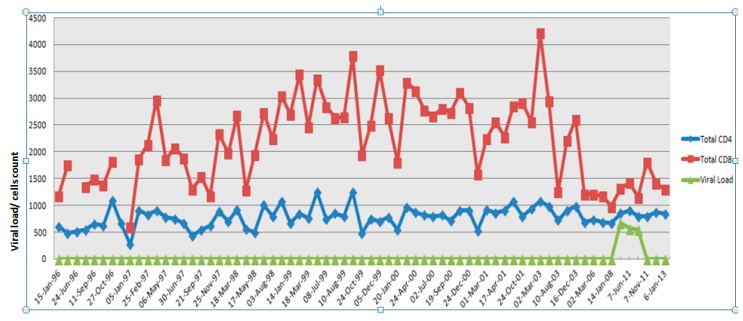
Summary of the study subject’s CD4^+^ and CD8^+^ T cell counts and plasma viral load between 1996 and 2013.

The blood samples were obtained from a 64-year-old male, an elite controller who was presumed infected with HIV in December 1988 through homosexual transmission from his partner, who died of AIDS on 22 January 1993. He was diagnosed seropositive by both ELISA and Western blot in 1993. The study subject has since remained asymptomatic, and plasma viral loads have remained undetectable (<50 copies/mL) since the first sample (1993) was tested. In May 2011 the subject had a transient viral blip that lasted for 4–5 months, where his viral load in plasma was recorded at 500–600 copies/mL. We termed this phase the viremic phase and 70 mL of patient blood was collected in 10 mL EDTA tubes on 18 May 2011 and on 7 June 2011. After this, the subject fully recovered and the viral load was below detectable levels (BDL) along with normal CD4^+^ and CD8^+^ T cell counts. We termed this the aviremic phase and collected 70 mL of blood on 7 November 2011. This work was approved by the Human Ethics Committee of Westmead Hospital. Figure 1 shows the summary of the study subject’s CD4^+^ and CD8^+^ T cell counts over time in relation to plasma viremia in the absence of HAART treatment.

Previously this patient has undergone several therapeutic trials. He was enrolled in a trial of Imiquimod (an IFN-α inducing agent) during 1992 [14]. He also received two sessions of whole-body 434-MHz microwave therapy in April and August 1992, in addition to the p24 virus-like particle (p24VLP) vaccine trial between January and June 1993–1994 [15]. The p24 vaccine trial was inconclusive as the patient developed adverse effects to Azidothymidine (AZT) mono-therapy and only received two of the six scheduled vaccine doses. The patient underwent further two courses of microwave therapy in 1994. During his visit to the clinic, a viral blip was discovered on 18 May 2011, which coincided with gallbladder (GB) inflammation; this sample was not analyzed in the study. Only upon monitoring inflammation and termination of treatment with steroids was another sample collected on 7 June 2011, at which time GB inflammation had subsided completely; the viral blip persisted in this sample. This was deemed appropriate for the protein analyses. Following this collection, the viral blip subsided completely and the patient has maintained below detectable levels of viremia and healthy lymphocyte counts subsequent and prior to the event in 2011 [12].

### 2.2. PBMCs Separation and Sample Preparation

Whole peripheral blood mononuclear cells (PBMCs) were extracted using Ficoll separation method. PBMCs were washed once with PBS, pelleted and were divided into two fractions. This step was repeated for both the viremic and the aviremic phases individually in order to assay p24 antigen-stimulated and un-stimulated fractions. Equal amount of cells were re-suspended for each fraction in RPMI medium 1640 containing 10% FBS in a non-adherent “Nunc^®^ dishes, low cell binding” to yield the maximum amount of cells. One fraction of PBMCs was stimulated with HIV capsid protein p24 at 2 ng/mL. This is the standard concentration used for assaying IFNγ production using Enzyme-linked immuno-sorbent spot (ELISPOT) assay. Both the un-stimulated and stimulated fractions were incubated overnight at 37 °C with 5% CO_2_.

### 2.3. Macs Column Separation of CD4^+^, CD8^+^ T Cells and Monocytes

Following overnight stimulation (12 h) of patient PBMCs from p24-stimulated and un-stimulated fractions were extracted by gently pipetting up and down to detach the cells from the non-adherent plate into 15 mL BD falcon tube. PBMCs were centrifuged at 1000 g to remove cell debris. Cell pellet was further washed twice with PBS to remove excess antigen. After aspirating all the PBS, the cell pellet was further washed with 10 mL of Magnetic cell-sorting (MACS) buffer (50 mL PBS + 250 µL of Fetal bovine Serum (FBS) + 200 µL of 2 mM EDTA) by centrifuging at 300× *g* for 10 min at 4 °C. Cells were washed with PBS in order remove any inherent cytokine/chemokine expression. A baseline was obtained using the un-stimulated fraction as both stimulated and un-stimulated cells were conditioned using the same method.

MACS^®^ Column Technology was used for separating PBMCs into different cell subsets. Using MACS^®^MicroBeads CD14^+^ monocytes, CD4^+^ helper T cells, and CD8^+^ cytotoxic T cells were extracted according to the manufacturer’s specifications using the positive selection Magnet Activated Cell Sorting method (MACS), Miltenyi Biotech (Marburg, Germany). CD14^+^ monocytes were extracted first to remove the by CD4^+^CD14^+^ T cell sub-population, followed by CD4^+^CD14^−^ helper T cells and CD8^+^ cytotoxic T cells. As per instructions, PBMC pellet was washed with MACS buffer and re-suspended in 80 µL of MACS buffer and 20 µL CD14^+^ beads per 10^7^ total cells. Cells were incubated for 15 min at 4 °C–8 °C re-suspended in 2 mL MACS buffer and spun at 300× *g* for 10 min to wash of the unbound/excess beads. Supernatant was pipetted completely and cells were re-suspended in 500 µL of MACS buffer. Magnetic separation was carried out with MS columns. Extracted cells were centrifuged for 10 min at 300× *g* to obtain a purified cell pellet and further washed with PBS. This process was repeated in the order of CD14^+^, CD4^+^CD14^−^ and CD8^+^ to extract cells. Flow cytometric verification of cell purity of CD4^+^, CD8^+^ T cells, and monocytes was evaluated using flow cytometry, as previously described by us [16].

### 2.4. Protein Extraction

Whole cellular proteins were extracted from both p24-stimulated and un-stimulated fractions of purified CD14^+^ monocytes, CD4^+^ helper T cells and CD8^+^ cytotoxic T-cells from each phase as per RayBiotech^©^ (Norcross, GA, USA) instructions. The 2×RayBio^®^Cell Lysis Buffer was diluted with H_2_O. 1:1 dilution of 150µL of 2× Raybio cell lyses buffer was used to lyse equal input of cells (5 × 10^6^) throughout our assays for protein extraction. One percent protease inhibitor (Sigma Aldrich, St Louis, MO, USA) was also added to prevent protein from degrading. Each of the samples was homogenized by sonication for one minute and the cell debris was removed by centrifuging at 10,000× *g* for 5 min. Lysates were stored at −80°C and were used within a week.

### 2.5. Quantification Using the BioRad DC-Protein Assay

A Bio-Rad DC-protein assay kit and the reagent pack (Bio-Rad, Gladesville, NSW, Australia) were used to quantify proteins as per manufacturer’s instructions. Five microliters of sample volume was used and 0.25, 0.5, 0.75, 1, and 1.5 mg/mL standards were used to get the standard curve. Bio RAD SmartSpec Plus spectrometer was used in measuring the absorbance at 750 nm. Final total protein concentrations in lysates of each cell type ranged from 1000 to1500 µg/mL, which were within the required analytical range for the protein array assay.

### 2.6. RayBio^®^ Combination of Human Cytokine Antibody Array G Series

Proteomic analysis of 274 cytokines, chemokines, their receptors, ligands and associating proteins from the CD4^+^, CD8^+^ T cells, and monocytes was carried out as per instructions from RayBio^®^ Combination of Human Cytokine Antibody Array G Series kit (Ref: AAH-CYT-G4000-8), which was purchased from RayBiotech^®^.

Glass slides were blocked with blocking buffer for 30 min. 50 µL of sample mixture (27 µg protein) from each of unstimulated and p24-stimulated fractions from each of the viremic and non-viremic phases were added to the sample wells along with 1 μL of Internal control and incubated overnight at 4 °C. After washing with buffers I and II, slides were incubated with biotin conjugated antibodies for 2 h at room temperature. Slides were washed and incubated for a further 1 h at room temperature in the dark with 70 µL of fluorescent dye conjugated Strepavidin. Slides were washed and dried by centrifugation at 1000 rpm for 3 min. Slides were scanned using an Axon Genepix scanner using the cy3 channel or at excitation frequency of 532 nm.

### 2.7. Data Normalization and Analysis

After normalizing the expression values as per instructions from RayBiotech^©^ using the positive controls, the fold-changes (FC) were obtained by comparing the un-stimulated cell fraction against the p24-stimulated cell fraction. This step was repeated for both phases and across all three cell-types to obtain the fold-change comparisons between the two phases.

As per instructions from RayBiotech, data were processed in Microsoft Excel 2007, where background was subtracted for the expression value to obtain the true expression. These protein expression values were then normalized according to the RayBiotech guidelines using the positive control from the reference array as the standard. The following equation was used to normalize the data between the two phases:

X(N*y*) = X(*y*) × P1/P(*y*), (1)

where P1 = the average signal expression value of the positive control spots on the reference array; P(*y*) = the average signal expression value of the positive control spots on Array *y*; X(*y*) = the signal expression value for a particular spot on Array for sample “y”; and X(N*y*) = the normalized value for that particular spot “X” on Array for sample “*y*”.

The fold-changes were obtained by dividing the expression values of the un-stimulated fraction by the p24-stimulated fraction for each corresponding protein. These fold changes were converted to log scale and a negative sign was given to depict a down-regulation.

### 2.8. Statistical Analysis Using K-Way and Log Linear Models to Discover K–Gene Interactions

We used a combination of association rule mining and log-linear modeling to discover k–gene interactions. Using this technique we could discover interactions among k-genes that cannot be explained by the combined effects of any of the subsets of those genes. Our results reveal some previously unknown associations between cell-types and proteins that point to solid biological explanations.

Further, the Log-linear analysis technique was used to examine the relationship between more than two categorical variables—in this case three-cell types CD4^+^, CD8^+^ T cells, and CD14^+^ monocytes and their encoded proteins. The technique was used for both hypothesis testing and possible model building. A Pearson’s chi-square test was used instead of log-linear analysis, but this only allowed for two of the variables to be compared at a time [17].

Log-linear analysis uses a likelihood ratio statistic *X*^2^: [18] that has an approximate chi-square distribution when the sample size is large:

(2) $$x^{2}=2\sum O_{\mathrm{ij}}\ln\frac{O_{\mathrm{ij}}}{E_{\mathrm{ij}}}$$

where ln = natural algorithm; *O_ij_* = observed frequency in cell*_ij_* (*i* = row and *j* = column); *E_ij_* = expected frequency in cell*_ij_*; and *X*^2^ = the deviance for the model [19].

### 2.9. Follow-up Tests

Once the model of best fit was determined, the highest-order interaction was examined by conducting chi-square analyses at different levels of one of the variables. For the chi-square analyses, it needed to break the model down into a 2 × 2 or 2 × 1 contingency table. For example, if one is examining the relationship among four variables, and the model of best fit contained one of the three-way interactions, one would examine its simple two-way interactions at different levels of the third variable. The results of log linear analyses are shown in Table 1 below.

## 3. Results

Our results are unique in showing the first snapshot of the length and breadth of cellular p24-antigen responses at the cellular and proteomic level for 274 cytokines, chemokines, their receptors, ligands, and associating proteins simultaneously in CD4^+^, CD8^+^ T cells, and monocytes of an HIV+ Long-term non-progressors (LTNP) at the viremic and aviremic phases after 20 years of receiving the HIVp24 vaccination. Proliferative responses to p24 antigen are still robust in this individual, as per our previous report [1,12]. In the current study, the data show evidence not only for the differential regulation of cytokines/chemokines and associating proteins in diverse leukocytes at viremic and aviremic phases in response to p24 antigen, but also their differential expression (DE) within the same cell type at these two disease phases in this individual.

As per manufacturer’s instructions, the fold-changes between >1.5 and <−1.54 were deemed non-significant. At the individual cell level, using this cutoff, the CD4^+^T cells yielded 59, monocytes 53, and the CD8^+^ T cells 52 DE proteins, respectively, from a total of 274 proteins analyzed on the protein array plate.

### 3.1. Overlapping Proteins that Functionally Bind CD4^+^, CD8^+^ T Cells,and CD14^+^Monocytes: Evidence for Crosstalk between Cell Types

**Figure 2:**
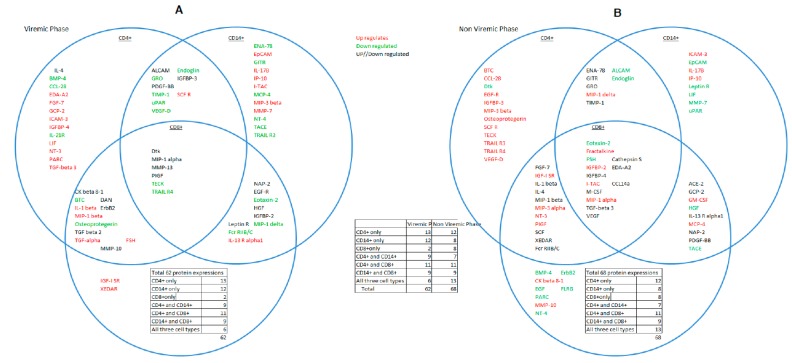
Venn diagram. Interaction and relationship between CD4^+^T, CD8^+^T cells, and CD14^+^ monocytes during the viremic (**A**) and aviremic phases (**B**). Insets within the circles show the DE genes and their breakdown within an individual cell type. Overlapping proteins between cell types are shown within the overlapping circles in figures (**A**) and (**B**).

Proteins that overlapped between all three cell-types were visualized first because of their coordinated response to p24 antigen, which has vital functional significance at different stages of HIV infection and secondly because a coordinated cellular response has not been previously visualized at the proteomic level. A combined analysis of all three cell-types showed 68 significant DE proteins in the aviremic phase and 62 in the viremic phase (Figure 2A,B).

Of 62 DE proteins at the viremic phase, six proteins—Dtk, MIP-1α, MMP-13, PIGF, TECK, and TRAIL R4—were overlapping between the three cell-types. Of these six proteins, four—Dtk, MIP-1α, MMP-13, and PIGF—expressed the same trend in two of the cell types but a reverse trend was observed in the other. However, two proteins, TECK and TRAIL-R4, showed similar expression trend in all three cell-types in this phase (Figure 2A,B). These critical differences in the expression of these proteins clearly segregated the viremic and aviremic phases, notably, the high-fold change (FC) in Dtk (17 FC), MMP13 (10 FC), and PIGF (−17 FC) in monocytes during the viremic phase. Moreover, the Dtk (−15-fold) and MIP-1α (nine-fold) also showed high expression in CD8^+^ T cells at viremic phase. This differential regulation of the same proteins in diverse cell types at the viremic phase is further suggestive of possible cross-talk between cell types in modulating the expression of these proteins at a given disease phase. Full summary of the 62 DE proteins in the viremic phase is given in Supplementary Table S1.

### 3.2. HIV Aviremic Phase

At the aviremic phase, based on all three cell-types, 14 proteins—Eotaxin-2, Fractalkine, FSH, Cathepsin-S, IGFBP-2 and 4, EDA-A2, IGFBP-4, I-TAC, CCL14a, M-CSF, MIP-1α, TGF-β-3, and VEGF—were differentially expressed. Of these 14 proteins, five in particular (Fractalkine, Cathepsin-S, CC14a, and IGFBP-2 and 4) showed systematic discriminatory expression trends between viremic and aviremic phases. Fractalkine, in particular showed consistently high fold-changes across all three cell-types, which has not been reported previously; these differences between viremic and aviremic phases further stress their functional significance in HIV disease. In contrast, the other nine proteins, although segregated between the two disease phases, appear to be regulated differentially between cell types. Chemokine MIP1-α was the only protein overlapping for all three cell-types and also for both phases of disease; it was up-regulated in all three cell-types with significant fold-change, except in monocytes during the viremic phase. Its highest up-regulation (9 FC) was seen in the CD8^+^ T cells during viremia. MIP-1α and MIP-1β have been shown to be increased in the LTNPs and are ligands for the CCR5 receptor [20]. I-TAC was up-regulated in all three cell-types but was unique in its high expression in CD4^+^ T (7 FC) and CD8^+^ T cells (13 FC) during aviremic phase, but its levels of expression were comparable in monocytes between the two phases. MCSF was significant for aviremia for all three cell-types, but was highly up-regulated (11 FC) uniquely in CD8^+^ T cells during aviremia.

The notable feature of these analyses was that during the aviremic phase the majority of responses emanated from the CD8^+^ T cells, followed by CD4^+^ T cells, which was evident from high fold-changes in the expression of seven of 11 overlapping proteins in this phase. This feature was consistent with the previously described role of CD8^+^ T cells in both non-progressive HIV disease and also in containing viremia. What is more significant about these proteins specific for viremic and aviremic phases is that such proteins have not been described in combination as shown here, and they may hold greater significance in guiding these disease phases (see the Supplementary Table S2 for the summary of 68 DE proteins at the aviremic phase).

### 3.3. Cell-Specific Differentially Expressed Proteins during Viremic and Aviremic Phases

#### 3.3.1. Proteins Segregating Viremic and Aviremic Phases Based on CD4^+^ T Cells

Apart from the coordinated response all three cell-types mounted (as discussed above), we also observed cell-specific responses discriminating between the viremic and aviremic phases of HIV infection.

For instance, in CD4^+^ T cells 59DE proteins showed significant fold-changes. Forty DE proteins were seen in the viremic phase, whereas 43 DE proteins were specific to the aviremic phase (Figure 2A). Of the 40 DE proteins for the viremic phase, DTK (−9.966 FC), Endoglin (−5.557), ICAM 3 (12.185), PIGF (4.513), IL21-R (−15.041), LIF (9.118), Osteoprotegerin (−15.166), TECK (−8.937), TGF-β3 (31.911), and VEGF-D (−29.987) showed high fold-change expression values. Whereas, of the 43 DE proteins for the aviremic phase, DTK (−4.916), Eotaxin-2 (−5.706), Fractalkine (14.720), FSH (6.718), IGFBP-2 (8.358), IL-4 (−7.651), I-TAC (7.138), and XEDAR (8.613) showed high fold-changes in expression (Figure 2A).

Another notable feature was the mirror regulation of certain cytokines, such as the up-regulation of EDA-A2, FGF-7, IL-4, IL-1β, IGFBP-4, and ALCAM proteins during viremic phase in the CD4^+^ T cells, and their down-regulation in the aviremic phase, suggesting their strong functional significance. Similarly, GRO, IGFBP-3, Osteoprotegerin, TECK, TIMP-1, CCL-28, BTC, VEGF-D, VEGF, and TRAIL R4 were up-regulated in the aviremic CD4^+^ T cells and were down-regulated in the viremic phase for the same cell type (Figure 2A,B).

Apart from this, some proteins were also significantly expressed in both phases in CD4^+^ T cells, displaying similar expression trends, but the differences in fold-changes of these proteins were actually robust in discriminating between viremic and aviremic phases, as evident from high expression of Dtk, Endoglin, and PIGF during the viremic phase and FSH, MIP-1α, MIP-1β, and TGF-β3 during the aviremic phase (Figure 2A,B).

**Figure 3:**
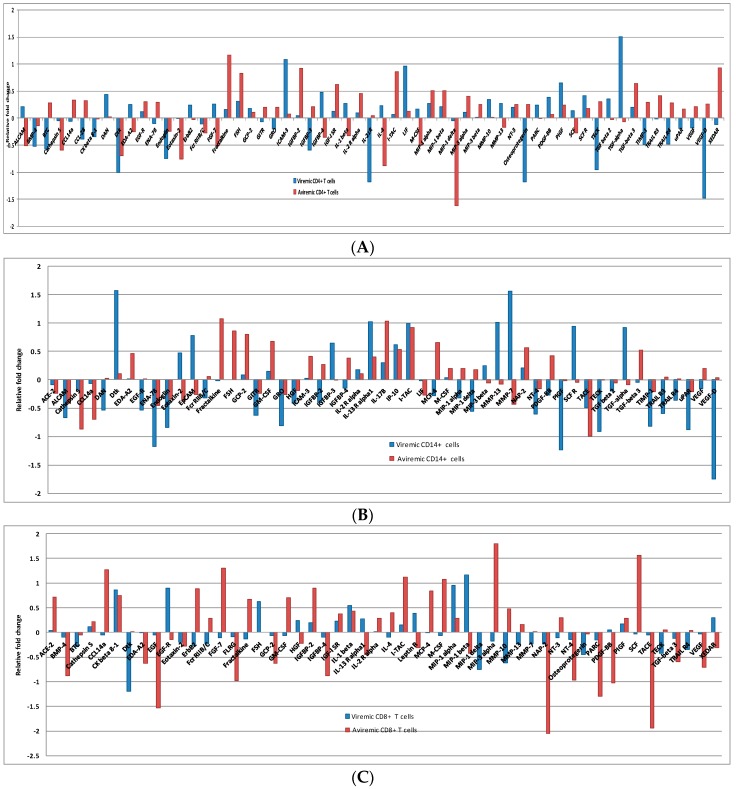
Log scale CD4^+^ (**A**), CD14^+^ (**B**), CD8^+^ (**C**) T cell foldchange during viremic and aviremic phases in log scale, where a negative log value has been shown to depict down-regulation in relation to up-regulation.

In the CD4^+^ T cells, the 59DE proteins showed significantly expressed fold-changes in both phases. Of 59 DE proteins, 40 were expressed in the viremic phase and 43 in the non-viremic phase (Figure 3A). Among the 40 DE proteins in the viremic phase ICAM 3 (12.185), IL21-R (−15.041), LIF (9.118), TECK (−8.937) and TGF-α3 (31.911) had very high fold-change expression values. Similarly, among the 43 fold-change expressions in the aviremic phase Eotaxin-2 (−5.706), Fractalkine (14.720), IGFBP-2 (8.358), MIP1-δ (41.3), I-TAC (7.138), and XEDAR (8.613) had very high fold- change expression values. Thus, there was a clear distinction between DE proteins expressed in aviremic *vs.* the viremic phase in CD4^+^ T cells—an observation not previously reported.

Again the notable feature was the mirror regulation of certain cytokines, such as the up-regulation of EDA-A2, FGF-7, IL-4, IL-1β, IGFBP-4, and ALCAM proteins during viremic phase in the CD4^+^ T cells, and their down-regulation in the aviremic phase (Figure 3A). Similarly, GRO, IGFBP-3, Osteoprotegerin, TECK, TIMP-1, CCL-28, BTC, VEGF-D, VEGF, and TRAIL R4 were up-regulated in aviremic CD4^+^ T cells and down-regulated in the viremic phase (Figure 3A). This reverse trend of expression was also observed in both CD14^+^ monocytes and CD8^+^ T cells, supporting their functional significance of mirror regulation of these cytokines/chemokines in modulating viremic and aviremic phases of HIV disease.

Also noteworthy is that some fold-changes in proteins were significantly expressed in both phases with a similar expression trend; however, this expression was very high only in one phase and lower in the other phase. This was evident with the expressions of Dtk, Endoglin, and PIGF with high fold-changes during the viremic phase and FSH, MIP-1α, MIP-1β, and TGF-β3 during the aviremic phase (Supplementary Table S3).

#### 3.3.2. Proteins Segregating Viremic and Aviremic Phases Based on CD14^+^ Monocytes

A total of 53 DE proteins were expressed in monocytes, with 40 in the viremic phase and 36 in the aviremic phase (Figure 3B). The most prominent proteins expressed in the viremic phase were DAN (−3.444), Dtk (37.758), EGF-R (−3.409), IGFBP-3(4.399), MMP-13 (10.3250), PIGF (−17.390), SCF R (8.777), TECK (−8.183), TGF-α (8.389), TIMP-1 (−6.598), TRAIL R3 (−3.902), and VEGF-D (−55.239). In contrast, the highly expressed proteins in the aviremic phase were fractalkine (11.945), FSH (7.178), GCP-2 (6.344), GM-CSF (4.715), CathepsinS (−7.38), and CCL14a (−5.04). The CD14^+^ monocytes showed up-regulation of proteins MMP-7, EPCAM, and IGFBP-2 in viremic phase and up-regulation of MIP-1δ, PDGF-BB, MCP-4, and MIP-1α in the aviremic phase. In addition to this, certain proteins were significantly expressed in both phases with a similar trend, although the degree of expression was higher only in one phase. This was observed with the expression of TIMP-1, ALCAM, ENA-78, Endoglin, GITR, GRO, IL-13 R α1, IP-10, I-TAC, and uPAR in the viremic phase and NAP-2, TACE, and IL-17c in the aviremic phase (Supplementary Table S4).

#### 3.3.3. *Proteins Segregating Viremic and Aviremic Phases Based on CD8^+^ T Cells*

Unlike the two other cell types, the expression fold-change in CD8^+^ T cells was more dominant in the aviremic phase. Of the 53 differentially-expressed proteins, 25 were expressed in the viremic phase and 42 in the non-viremic phase (Figure 3C and Supplementary Table S5). For instance, the Dtk (−15.493), EGF-R (7.874), FSH (4.195), and MIP-1 δ (−5.584) were notable in the viremic phase with their high-fold change expressions, while ACE-2 (5.191), BMP-4 (−7.524), CCL14a (18.690), EDA-A2 (−4.202), EGF (−33.681), FGF-7 (20.007), FLRG (−9.492), Fractalkine (4.651), GCP-2 (−4.230), GM-CSF (5.085), IGFBP-4 (−7.524), I-TAC (13.259), MCP-4 (6.9474), M-CSF (11.791), NT-4 (−9.349), PARC (−19.778), PDGF-BB (−10.598), SCF (36.281), TACE (−86.264), TGF-β 3 (−3.977), and VEGF (−5.110) were specific to the aviremic phase. The reverse trend was observed in CD8^+^ cytotoxic T-cells with MIP-1β, HGF, Leptin R, XEDAR, and IL-13Rα, being up-regulated in the viremic phase and down-regulated in the aviremic phase. Moreover, the mirror regulation was also noted for the proteins MIP-3g, Fcr RIIB/C, MMP-10 and ErbB2, with up-regulation during the aviremic phase (Figure 3C).

### 3.4. CD4^+^ T Cells and Monocytes Are Key Players during Viremia, Whereas CD4^+^, CD8^+^ T Cells and Monocytes Are Equal Partners during the Aviremic Phase

In this analysis, we teased out where the most immune responses during viremic and aviremic phases are emanating from. For this, we compared side-by-side all three cell types for the differentially-expressed proteins and calculated the percentages of proteins representing each cell type at a given phase of each cell type. These analyses showed that CD4^+^ T cells and monocytes mounted the greatest response during viremia, with 40/62 DE proteins (68%) each for monocytes and CD4^+^ T cells involved in this response, as opposed to 25/62 (40%) of DE proteins from CD8^+^ T cells. This suggests that although all three cell-types have a significant role during viremia, the dominant partners were the CD4^+^ T cells and monocytes. In contrast, during aviremia the immune responses mounted by CD8^+^ T cells were much higher than observed in viremic phase, consistent with their cytotoxic role and in non-progressive HIV disease, with 42/68 DE proteins (62%) involved in this response. But when CD8^+^ T cells were compared against CD4^+^ T cells (63%) and monocytes (53%), it was apparent that all three cell-types were dominant partners during aviremia, which has never been shown before at the protein level and signifies functional cross-talk between cell-types in controlling viremia (Supplementary Table S6).

### 3.5. Q-RTPCR Validation of Proteomic Expression: High Concordance between Protein and Genomic Expression Trends

In order to validate the functional value of these proteins, we attempted a reverse validation (protein to gene). Using q-RTPCR, we performed genomic validation of six DE proteins—MIP-1β, Eotaxin-2, IP-10, I-TAC, MIG, and MCP-2—by designing appropriate gene-specific primers. We observed a consistent trend of expression between our proteins and the genomic data (Table 2) with the exception of protein MIP-3β, which showed a reverse expression trend consistent with previous observations that protein expression does not always correlate with gene expression, as some genes are only transcriptionally active or *vice versa*. This in no way changes the interpretation of this study.

### 3.6. K-Way and Log-Linear Model Analysis Reveal Joint Expression of Genes from All Three Cell Types Significant in Two- and Three-Way Interactions

The k-way and log linear model analysis solidifies one critical observation: that proteins tend to express in pairs or all three of CD8, CD4, or monocyte rather than with only a single one of these. Overall we observed far fewer proteins expressing just only of CD8, CD4, or monocytes than would be expected if these act independently (*p* < 0.001). Instead we observed many more proteins than expected expressing none of CD8, CD4 or monocyte, or two or three of these simultaneously (chi-square *p* < 0.001). In the log linear model of the joint expression of genes with CD8, CD4, and monocytes, all two-way and three-way interactions were significant (*p* < 0.001 for each interaction, see Table 1). Thus, our observations on coordinated cellular response by all three cell-types in a given phase of HIV disease are unambiguously supported by these statistical analyses.

## 4. Discussion

In this study we examined HIV-1-specific immune responses at the level of 274 cytokines, chemokines, their receptors, ligands, and associated proteins post-immunization with an HIV-1 immunogen p24 in a rare elite controller. Since immune responses against HIV-1 Gag are known to be associated with improved control of HIV infection [21], it is believed that Gag-like peptides may be good candidates for an effective therapeutic vaccine. Even though peptide-specific cellular immune responses were induced in the study subject, the protective correlates of effective immunity to p24 have never been defined in such detail as attempted in this study using antibody arrays. This could partly be attributed to the p24 vaccination trial being inconclusive; no follow-up was done due to the failure of the vaccine. The continued presence of robust proliferative responses and strong cellular responses to p24 antigen post-vaccination in the study subject prompted this study to holistically define cellular responses by visualizing them at the protein level.

This rare HIV+ non-progressor who received the p24 vaccine in 1993 continues to live disease- and viremia-free in the absence of HAART therapy with high CD4^+^ T cell counts (700/µL blood taken in January, 2013) [1,12]. Moreover, the continued presence of p24 responses after 20 years of p24 vaccination raises the possibility that p24 vaccination in this individual may have worked, although we do not have pre-vaccination data on p24 responses dating back to 1992. The main stimulus to carry out such a detailed study of p24 antigen-induced cellular responses at the protein level emanates from a notable occurrence of a rare plasma viremia blip (563 HIV RNA copies/mL plasma) in 2011, which naturally subsided within a few weeks without therapeutic intervention. Although his viral blip coincided with gallbladder inflammation, we discarded the first sample collected close to the gallbladder inflammation (18 May 2011) and used the second sample collected (7 June 2011) when the inflammation subsided upon treatment. This allowed us a rare window of opportunity to study cellular immune responses uniquely related to the p24 antigen in both the viremic and aviremic phases from blood samples collected in 2011. Our comprehensive and simultaneous analyses of cellular responses to p24 antigen in the patient’s CD4^+^, CD8^+^ T cells, and monocytes provided the first snapshot of the protective immune correlates of p24 vaccination. The notable finding is that the proteins associated with coordinated cellular responses and cell-specific immune responses clearly discriminated between proteins associated with the viremic and aviremic phases of HIV disease.

Through these analyses, we were not only able to show cohesive and intimate interaction between CD4^+^, CD8^+^ T cells, and CD14^+^ monocytes, but were also successful in deciphering the proteomic basis of the broad crosstalk between these cell-types that segregated these two phases. The proteins—Dtk, MIP-1α, MMP-13, PIGF, TECK, and TRAIL-R4—were specific for the viremic phase, whereas the proteins Eotaxin-2, Fractalkine, FSH, IGFBP-2 and 4, Cathepsin-S, EDA-A2, I-TAC, CCL14a, M-CSF, MIP-1α, TGFβ3, and VEGF were specific to the aviremic phase. Many of these phase-specific proteins have not been previously described in the context of HIV disease staging; our data sheds a new light on their role in guiding viremic and aviremic phases through coordinated regulation across cell types. Further, apart from the coordinated immune responses from all three cell-types, some of the proteins were highly expressed in two of three or one of three cell-types analyzed, suggesting that variable expression trends between cell types had less bearing on the outcome in controlling viremia or aviremia. It was the coordinated immune response and cross-talk between cell-types that was vital in providing functional synergy and guiding the quality of the immune response required in a given phase of HIV disease.

Both for the viremic and aviremic phases, many of the cytokines, chemokines, their receptors, ligands, and associating proteins shown here have not been shown before, because most previous studies have analyzed a single cytokine or a few cytokines at best. Analyzing proteins in an antibody array format has provided a new paradigm in understanding and visualizing novel proteins involved in HIV-specific cellular responses that may prove to be important in future vaccine strategies for HIV. One significant example is Fractalkine (CX3CL1), which is a low-molecular-weight protein and is the unique ligand for the chemokine receptor CX3CR1 expressed on monocytes, natural killer cells, and T cells [22] and is involved in chemotaxis, adhesion of leukocytes, and proliferation [22,23]. Previously, increased levels of Fractalkine in the cerebrospinal fluid have also been implicated in the progression of dementia in HIV-positive patients [24]. However, recent publications have shown that Fractalkine may play a role in promotion of cell survival during homeostatic and inflammatory conditions [25,26]. In our study we observed Fractalkine levels were up-regulated in all three cell types throughout the aviremic phase, suggesting its protective effect, which is consistent with the studies by Landsman *et al.* [25] and Karlmark *et al.* [26]. We believe that the protective effect of Fractalkine could also be mediated by its interaction with partner proteins (Cathepsin-S, CCL14a, and IGFBP-2 and 4) during the aviremic phase, but it remains to be proven how these proteins work in concert. Further, we also found that during the aviremic phase antiviral, pro-inflammatory, and anti-apoptotic cytokines were up-regulated, suggesting that the host is able to mount an effective T-cell-derived immunity without leading to T cell exhaustion. Thus, it is plausible to hypothesize that the up-regulation of proteins inducing a protective effect during aviremic phase are possibly vital for maintaining healthy T-cell populations and any imbalance or deterioration in the immunological health of any of the cell types may tilt this balance in favor of viremia—an aspect reminiscent of HIV disease.

Our study showed increased levels of ICAM 3 (intercellular adhesion molecule 3) expression in CD4^+^ helper T cells during the viremic stage, which is in accordance with previous findings [10,27] where increased ICAM3 is associated with plasma viremia. As a protein involved in co-stimulation, increased levels of ICAM-3 have not only been shown to increase HIV-1 transcription and viral production, but also to augment the infection of resting CD4^+^T cells [10]. We observed a 12-fold increase of ICAM3 expression in CD4^+^ T cells at the viremic stage, consistent with its involvement of co-stimulatory mechanisms in viral entry.

The natural ligands for CCR5 are macrophage inflammatory protein 1α and 1β (MIP-1α and MIP-1β), human CC chemokine ligand 3 like 1 gene (CCL3L1), and RANTES and the only ligand for CXCR4 is SDF-1 [28]. These natural chemokine ligands are known to act as competitive inhibitors of HIV-1 during viral entry and to down-regulate the expression of these co-receptors on the cell surface, thereby becoming associated with the control of HIV1 disease progression [28,29]. CD8^+^ T cells and macrophages secrete most of the aforementioned chemokines, while RANTES is known to be secreted by platelets [28,30]. In this study we observed down-regulation of both MIP-1α and MIP-1β expression in the viremic phase CD4^+^ T cells and CD14^+^ monocytes when compared to the aviremic phase. However, in CD8^+^ T cells MIP-1α and MIP-1β expressions were highly up-regulated in the viremic phase compared to the aviremic phase, suggesting that CD8^+^T cells play a crucial role in viral suppression and highlighting their significance in viremia control during HIV infection. In genomic validation of these findings, we noted a similar expression trend for MIP-1α and MIP-1β, but it was also notable that even though MIP-1β was up-regulated in both phases in CD4^+^ T cells, its higher up-regulation at the aviremic phase carried much more functional weight and is consistent with their antiviral properties. Similarly, patterns were also observed for others in the macrophage inflammatory protein (MIP) family. Even though the cumulative relevance of cytokines and their associating proteins has never been evaluated in the viremic and aviremic phases, we believe that these proteins work in concert and play an important part in the homeostasis of these cell-types.

MIP-1δ/CCL15 is the ligand for chemokine receptors like CCR1 and CCR3 and is a known chemo-attractant for neutrophils, monocytes, and lymphocytes. Downregulation of MIP-1δ was observed in the aviremic CD4^+^ T cells and viremic CD14^+^ monocytes and CD8^+^ T cells. Up-regulation was observed in the aviremic CD14^+^ monocytes. Other inflammatory chemokines and recruiters of lymphocytes [31] of the MIP family such as CCL20/MIP-3α and CCL19/MIP-3β were also significantly expressed in both our studies. CCL20/MIP-3α was up-regulated in the aviremic CD4^+^ T cells and viremic CD8^+^ T cells, and also detected in aviremic CD8^+^ T cells. Similarly, CCL19/MIP-3β up-regulation in both phases in all three cell-types was also consistent with the genomic dataset (Table 2). This trend was also observed in the aviremic CD4^+^ T cells and in viremic monocytes. MIP-3β is known to regulate CD4^+^ T-cell immune responses in the secondary lymphoid organs by promoting activation-induced cell death [32]. The effect of MIP-3β is two-fold as, in the presence of MIP-3β, antigen-stimulated CD4^+^ T cells are highly activated and MIP-3β also promotes activated T cell differentiation into memory T cells [32]. Further to this theory, Kim *et al.* [33] have shown that CCR7, a receptor associated with MIP-3β, protects CD8^+^ T cells from apoptosis.

The multiple roles played by CD4^+^ T cells during HIV infection as well as the fact of them being the prime target of HIV make them one of the most effective immune cells in dealing with the invading virus [34]. The cytotoxic potential of antiviral CD4^+^ T cells is critical for maintaining the homeostasis of the CD8^+^ T cells and antibody-producing B cells. It is known that many cytokines such as IFN-γ (interferon γ), IL-2, and Tumor Necrosis Factor α (TNFα) are produced by CD4^+^ T cells [34]. They are the first indicators of T-cell exhaustion, which leads to impaired function of CD8^+^ T cells and progressive loss of IL-2 production, followed by the loss of TNFα production [34]. Therefore, the loss of function of CD4^+^ T cells due to infections, such as seen in HIV/AIDS, is largely responsible for the impairment of antiviral immunity in the HIV-infected host, which possibly also causes an imbalance in the coordinated immune response with its partner cells. The increased IL4 to INF-γ ratio has been correlated with high levels of plasma viremia and rapid loss of CD4^+^ T cells [8]. In CD4^+^ T cells, we observed an up-regulation of IL4 during the viremic phase and a 7.5-fold down-regulation during the aviremic stage. However, we did not observe a significant expression of INFγ, which was quite stable in both phases in this patient.

CXC chemokine ligand 9 (CXCL9)/MIG, chemokines CXCL10/IFN-inducible protein 10 (IP-10), and CXCL11/IFN-inducible T cell α chemoattractant (I-TAC) are chemokines belonging to the IFN-2 inducible family. These chemokines are known to attract and activate CXCR3-bearing cells such as peripheral memory T, B and, natural killer cells, and *in vitro* T helper type 1 (Th1) cells, but not naive T cells [35,36,37]. These three chemokines have an antagonistic activity on CCR5 and may counteract the action of inflammatory chemokines such as MIP-1α that act via CCR5 [38]. Similarly, it is known that increased expression of these chemokines promotes recruitment of susceptible T cells, which in turn might enhance the sequestration of T cells in infected lymphoid organs and the spread of infection between cells, contributing to the immunopathology of AIDS [39].

Interestingly, IP-10 was up-regulated in all three cell-types in both phases, but this up-regulation was more prominent in viremic CD4^+^ T cells, CD14^+^ monocytes and in aviremic phase CD8^+^ T cells. Not much is known about MIG, I-TAC, and IP-10 in the context of HIV. However, a recent paper by Lajoie *et al.* [40] has shown that HIV-1-exposed seronegative (HESN) individuals have significantly lower expression of MIG and IP-10 in their genital mucosa compared with HIV-infected commercial sex workers. This is quite significant: since both LTNPs and HIV exposed but seronegative (HESN) individuals are able to control the infection naturally, they may express a similar cytokine profile; given the potential of these two chemokines, the down-regulation of these chemokines during the aviremic phase may offer some protective effect.

Along with MIG, IP-10, and I-TAC, five other chemokine antagonists including MIP-1β and Eotaxin-2 are known in the literature [39]. Other than MIP-1β, the functional relevance of the other proteins is not known in the context of HIV, but it would be interesting to see how these chemoattractant proteins in concert play a part in the activation and recruitment of lymphocytes during HIV infection. Another suggestion would be that the up-regulation of chemokine antagonists may be involved in the TH^1^ to TH^2^ shift, thereby favoring pro-viral activity or better regulation of antiviral cytokines to prevent lymphocyte recruitment and activity during viral entry. Both Eotaxin 1 and 3, which are known chemoattractants [41,42,43], were stable in both phases; however, Eotaxin-2 was down-regulated in the aviremic phase in all three cell-types and in viremic CD14^+^ and CD8^+^ T cells. This pattern was mirrored in the genomic data as well, where it was down-regulated in all three cell types in both phases, suggesting its vital functional role. However, its down-regulation was much more prominent in the viremic phase. MCP-4 (Monocyte chemotactic protein-4) was up-regulated in both CD14^+^ and CD8^+^ during the aviremic phase, while it was down-regulated in CD14^+^ in the viremic phase. MCP-4 shares 56%–61% sequence identity with the three known mono cyto-chemotactic proteins including MCP-3 (a known chemokine antagonist for CCR5 receptor) and is 60% identical with Eotaxin. MCP-4 is functionally similar to both Eotaxin and MCP-3. MCP-4, like MCP-3, is a chemoattractant of high efficacy for monocytes and T lymphocytes [44].

It needs to be iterated that HIV disease progression is due, in part, to accelerated rates of apoptotic cell death of infected, as well as non-infected bystander cells [9,45]. One of the main proteins involved in this apoptosis process is TRAIL (TNF-related apoptosis inducing ligand) as well as its cognate receptors [45]. TRAIL is a member of the TNF superfamily with ligands that include Fas ligand [46] (Huang *et al.* [47] 2006). TRAIL has five known receptors, surface expressed TRAIL-R1 through TRAIL-R4 and the soluble Osteoprotegerin. Two of the receptors, TRAIL-R1 and TRAIL-R2, are known inducers of apoptotic signal, while TRAIL-R3, TRAIL-R4, and osteoprotegerin are known to act as decoy receptors [46].TRAIL-R3 and TRAIL-R4 lack the intracellular regions necessary for signal transduction, while osteoprotegerin, a receptor heavily involved in bone remodeling, acts as a soluble inhibitor of RANK ligand, thereby preventing the apoptotic signal [46,48,49]. We saw a reverse trend between TRAIL-R3, TRAIL-R4, and Osteoprotegerin in CD4^+^ T cells, where their expression was down-regulated in the viremic phase but up-regulated in the aviremic phase. In fact, TRAIL-R4 levels were down-regulated throughout the viremic stage in all three cell types. TRAIL-R3 was also down-regulated in CD14^+^ T cells during the viremic phase, while Osteoprotegerin was down-regulated throughout the viremic phase in CD8^+^ T cells. HIV-associated T cell depletion is mediated, at least in part, by disordered apoptosis [9]. This patient seems to efficiently regulate TRAIL-mediated apoptosis and maintain the CD4^+^ T cell levels by utilizing TRAIL-R3, TRAIL-R4, and, to some degree, Osteoprotegerin. This protective effect may be due to the increased expression of TRAIL-R3, TRAIL-R4, and Osteoprotegerin during the aviremic phase. However, during the viremic phase the down-regulation of these receptors seems to coincide with depletion of CD4^+^ T cells, which has not been shown before. This also highlights that simultaneous modulation of these proteins at any given phase is more important functionally than the modulation of a single protein *in vivo* during HIV infection.

The proposal that protective cytokines play a major role in maintaining this patient’s T cell levels during the course of the infection is further reinforced by the fact that lesser-known chemokines such as TECK/CCL25 were down-regulated in the viremic phase in all three cell types, while being up-regulated during the aviremic phase in monocytes. This observation coincides with results from macaques infected with Simian immunodeficiency virus (SIV), where a decrease in TECK expression was associated with increased apoptosis in lymphoid tissues [50], suggesting that dysfunction in anti-apoptotic chemokines might be a mechanism that contributes to loss of immune function following pathogenic HIV infection. TECK (thymus-expressed chemokine) is known to play a role in the development of T-cells and mucosal immunity in mice [51,52].

Overall, this study is unique in providing the first snapshot of immune correlates of HIV-p24 antigen responses and their simultaneous analysis in CD4^+^, CD8^+^ T cells, and monocytes in a rare therapy-naïve HIV+ LTNP, who underwent p24 vaccination for HIV. Our analyses show for the first time how these immune correlates discriminate between viremic and aviremic phases at the level of broad cellular and cell-specific responses. These analyses have also shown that, although the CD8^+^ T cells play a significant role in aviremia, the equal participation of monocytes and CD4^+^ T cells is also needed. In contrast, the CD4^+^ T cells and CD14^+^ monocytes played an important role in the viremic phase in terms of the pro-inflammatory cytokine expression. These analyses are valuable in shedding light on mechanisms that regulate the immune homeostasis, especially in the aviremic phase of CD4^+^ and CD8^+^ T cells and CD14^+^ monocytes, and showing how the protective effect of anti-apoptotic proteins, evident during the infection with an up-regulation of these factors in the aviremic phase and a down-regulation during the viremic phase, is significant in modulating HIV disease stages. Overall, these analyses stress that a coordinated effort of three cell types and the crosstalk between them may be vital in functionally guiding a potent immune response at a given HIV disease phase.

## 5. Conclusions

In conclusion, our data provide the functional significance of the multifaceted simultaneous innate and adaptive immune responses to p24 antigen in CD4^+^, CD8^+^ T cells, and monocytes during viremic and aviremic phases of a rare HIV+ therapy naïve non-progressor, who received p24 vaccination. Along with providing the proteomic basis of cellular immune response that may have come into play post-p24 vaccination, understanding its impact on cellular defense system is sorely needed for future HIV vaccines. High-throughput technologies will allow for in-depth analysis and holistic understanding of immune responses that play a vital role during HIV disease. These analyses will pave the way for the development of novel approaches, which need integration in future vaccine strategies. It will be particularly critical to harness key innate immune mediators that contribute to control of viremia in HIV-infected individuals. Although we have analyzed only three cell types for evaluating cellular immune response to p24 antigen, the immune responses prompted by other cell types, such as NK cells and B cells, which also play a vital role in HIV infection, cannot be understated. More work is needed to createa global snapshot of HIV-specific cellular immune responses.

## Figures and Tables

**Table 1:** *K*-way and log-linear model statistical analyses.

*K*-Way and Higher-Order Effects	*K*	*df*	Likelihood Ratio Chi-Square	Sig.	Pearson Chi-Square
***K*-way and Higher Order Effects ^a^**	1	7	616.1	0	1056.511
	2	4	104.972	0	147.811
	3	1	22.957	0	25.08
***K*-way Effects ^b^**	1	3	511.128	0	908.7
	2	3	82.015	0	122.731
	3	1	22.957	0	25.08

^a^ Tests that *K*-way and higher order effects are zero. ^b^ Tests that *K*-way effects are zero. Monocyte than would be expected if these act independently (*p* < 0.001); Instead we observed many more proteins than expected expressing with none of CD8, CD4, or monocyte, or with two or all three of these simultaneously (chi-sq, *p* < 0.001); In the log linear model of the joint expression of genes with CD8, CD4, and monocyte, all two-way and three-way interactions were significant (*p* < 0.001 for each interaction, see below).

**Table 2:** Expression trends of co-expressed immune modulators between the qRTPCR and protein array for all three cell-types at viremic and aviremic phases.

Cytokines	PCR Array Expression						Protein Array Expression					
	Viremic CD4^+^	Aviremic CD4^+^	Viremic CD14^+^	Aviremic CD14^+^	Viremic CD8^+^	Aviremic CD8^+^	Viremic CD4^+^	Aviremic CD4^+^	Viremic CD14^+^	Aviremic CD14^+^	Viremic CD8^+^	Aviremic CD8^+^
CCL18/PARC	**156.28**	–	–	–	–	–	**1.72**	–	–	–	–	−19.78
CCL19/MIP-3B	5.42	**14.05**	**14.89**	47.90	12.06	10.93	–	**1.77**	**1.76**	–	–	–
CCL24/eotaxin-2	−6.46	**−6.81**	**−20.45**	**−4.19**	**−44.26**	**−7.52**	–	**−5.71**	**−2.96**	**−2.37**	**−1.58**	**−1.91**
CCL4/MIP-1- β	**5.97**	**6.16**	–	–	–	–	**1.63**	**3.23**	–	–	14.75	−2.67
CCL5/RANTES	–	–	−107.00	**16.94**	–	–	–	−1.76	–	**2.73**	–	–
CCL8/MCP-2	199.19	39.18	**68.40**	**25.49**	**18.40**	50.56	–	–	**2.49**	**12.47**	**1.71**	–
CD80	–	–	**4.38**	**4.88**	–	–	–	–	**1.53**	**3.79**	–	–
CXCL10/IP-10	83.17	16.13	**31.04**	**18.40**	67.74	15.89	–	–	**4.17**	**3.46**	–	–
CXCL11/I TAC	180.77	**66.81**	**78.58**	**103.39**	311.27	**41.93**	–	**7.14**	**9.88**	**8.26**	–	**13.26**
CXCL13/BLC	**6.05**	**26.03**	−5.29	–	4.41	**8.69**	**2.32**	**5.90**	1.54	–	–	**1.55**
CXCL2/GRO	**−10.72**	–	**−29.94**	**−7.20**	−13.34	–	**−1.58**	1.59	**−6.47**	**−1.82**	–	–
CXCL5/ENA-78	−205.36	−17.36	**−1041.18**	**−37.22**	−14.91	−18.64	–	1.98	**−14.91**	**−2.82**	–	–
CXCL9/MIG	79.23	28.29	**11.52**	**43.77**	94.48	34.06	–	–	**3.44**	**1.61**	–	–
IL8	–	–	**−11.66**	–	–	–	–	1.55	**−2.24**	–	1.57	–
VCAM1	32.85	15.89	**109.29**	60.38	–	–	–	–	**1.55**	–	–	–

NB: FC expressions left blank represent no significantly expressed genes/proteins. For the PCR, array genes with ±4 fold changes were deemed significant as per the manufacturer’s instructions. For the protein array, protein fold changes ≤−1.53 and ≤1.5 were deemed significant as per the manufacturer’s instructions. Bold fold change represent trends that are akin in both arrays, whereas normal fold changes represent immune modulators that are significantly expressed only in one array or when the trends are not matching.

## Supplementary Materials

The supplementary materials are available online at http://www.mdpi.com/2076-3905/5/2/14/s1.
