# Supplementary material for: Retrospective Proteomic Analysis of Cellular Immune Responses and Protective Correlates of p24 Vaccination in an HIV Elite Controller Using Antibody Arrays
Source: Microarrays (Basel). 2016 Jun 2;5(2):14. doi: 10.3390/microarrays5020014 (PMC5003490; doi:10.3390/microarrays5020014)
Supplement: Supplementary file 1 [file microarrays-05-00014-s001.pdf]

Supplementary Materials: Retrospective Proteomic Analysis of Cellular Immune Responses and Protective Correlates of p24 Vaccination in an HIV Elite Controller Using Antibody Arrays

Suneth S. Perera, Bin Wang, Arturo Damian, Wayne Dyer, Li Zhou, Viviane Conceicao, and Nitin K. Saksena

Table S1. Showing the 62 differentially expressed proteins in the viremic phase.

	Viremic Phase		
**Cytokines**	**CD4+ T Cells**	**CD14+ Monocytes**	**CD8+ Cytotoxic T Cells**
ALCAM	1.617	−4.629	–
BMP-4	−3.301	−1.491	–
BTC	−3.714	–	−1.967
CCL-28	−2.456	–	–
CK β 8-1	−2.201	–	7.248
DAN	2.753	−3.444	–
Dtk	−9.966	37.758	−15.494
EDA-A2	1.786	–	–
EGF-R	–	−3.410	7.874
ENA-78	–	−14.912	–
Endoglin	−5.574	−6.882	−1.512
Eotaxin-2	–	−2.956	−1.577
EpCAM	–	5.997	–
ErbB2	1.764	–	−1.842
Fcr RIIB/C	–	−2.070	−1.724
FGF-7	1.840	–	–
FSH	2.078	–	4.195
GCP-2	1.509	–	–
GITR	–	−4.255	–
GRO	−1.581	−6.474	–
HGF	–	−2.608	1.729
ICAM-3	12.185	–	–
IGFBP-2	–	−2.510	1.572
IGFBP-3	−3.942	4.399	–
IGFBP-4	3.012	–	–
IGF-I SR	–	–	1.687
IL-1 beta	1.880		3.472
IL-13 R α1	–	10.584	1.902
IL-17B	–	1.988	–
IL-21R	−15.041	–	–
IL-4	1.693	–	–
IP-10	–	4.174	–
I-TAC	–	9.875	–
Leptin R	–	−1.764	2.432
LIF	9.118	–	–
MCP-4		−1.913	–
MIP-1α	1.872	−2.083	9.036
MIP-1β	1.630	–	14.747

Table S1. *Cont.*

Viremic Phase			
**Cytokines**	**CD4+ T Cells**	**CD14+ Monocytes**	**CD8+ Cytotoxic T Cells**
MIP-1 delta	–	−3.609	−5.584
MIP-3β	–	1.757	–
MMP-10	2.216	–	−4.106
MMP-13	1.871	10.326	−1.717
MMP-7	–	36.798	−1.713
NAP-2	–	1.621	−1.730
NT-3	1.600	–	–
NT-4	–	−4.015	–
Osteoprotegerin	−15.166	–	−2.767
PARC	1.724	–	–
PDGF-BB	2.451	−1.875	–
PIGF	4.513	−17.390	1.506
SCF R	2.579	8.777	–
TACE	–	−3.137	–
TECK	−8.937	−8.184	−2.631
TGF β 2	2.250	−1.576	–
TGF-α	31.912	8.390	–
TGF-β 3	1.580	–	–
TIMP-1	−3.045	−6.598	–
TRAIL R3		−3.902	–
TRAIL R4	−2.972	−2.322	−2.235
uPAR		−7.542	–
VEGF-D	−29.987	−55.239	–
XEDAR	–	–	1.977

Table S2. Showing the 68 differentially expressed proteins in the aviremic phase.

Aviremic Phase			
**Cytokines**	**CD4+ T Cells**	**CD14+ Monocytes**	**CD8+ Cytotoxic T Cells**
ACE-2	−1.278	−1.727	5.192
ALCAM	−3.202	−2.591	–
BMP-4	–	–	−7.525
BTC	1.912	–	–
Cathepsin S	−3.920	−7.379	1.661
CCL14a	2.152	−5.038	18.691
CCL-28	2.086	–	–
CK β 8-1	–	–	5.636
Dtk	−4.916	–	–
EDA-A2	−1.802	2.937	−4.202
EGF	–	–	−33.681
EGF-R	2.016	–	–
ENA-78	1.981	−2.820	–
Endoglin	−1.743	−2.175	–
Eotaxin-2	−5.706	−2.366	−1.906
EpCAM	–	−1.820	–
ErbB2	–	–	7.565
Fcr RIIB/C	−1.867	–	1.921
FGF-7	−3.224	–	20.008

Table S2. *Cont.*

Aviremic Phase			
**Cytokines**	**CD4⁺ T Cells**	**CD14⁺ Monocytes**	**CD8⁺ Cytotoxic T Cells**
FLRG	–	–	−9.493
Fractalkine	14.720	11.945	4.651
FSH	6.719	7.178	
GCP-2	–	6.344	−4.300
GITR	1.601	−1.747	–
GM-CSF	–	4.715	5.086
GRO	1.594	−1.821	–
HGF	–	−1.541	−1.672
ICAM-3	–	2.574	–
IGFBP-2	8.358	1.859	7.907
IGFBP-3	1.627	–	–
IGFBP-4	−2.255	2.436	−7.525
IGF-I SR	4.184	–	2.393
IL-1 beta	−2.110	–	2.710
IL-13 R α1	–	2.502	−2.790
IL-17B	–	10.870	–
IL-4	−7.652	–	2.481
IP-10	–	3.460	–
I-TAC	7.138	8.256	13.260
Leptin R	–	–	−2.041
LIF	–	−1.853	–
MCP-4	–	4.556	6.947
M-CSF	−3.117	1.572	11.791
MIP-1 α	3.193	1.580	1.947
MIP-1 β	3.229	–	−2.670
MIP-1 delta	−41.333	1.509	–
MIP-3 α	2.578	–	62.329
MIP-3 β	1.768	–	–
MMP-10	–	–	3.016
MMP-7	–	−2.681	–
NAP-2	–	3.705	−112.906
NT-3	1.807	–	2.004
NT-4		–	−9.350
Osteoprotegerin	1.769	–	–
PARC	–	–	−19.779
PDGF-BB	–	2.636	−10.599
PIGF	1.744	–	1.947
SCF	−1.861	–	36.281
SCF R	1.502	–	–
TACE	–	−9.879	−86.264
TECK	2.001	–	–
TGF-β 3	4.391	3.332	−3.977
TIMP-1	1.946	−1.618	–
TRAIL R3	2.603	–	–
TRAIL R4	1.912	–	–
uPAR	–	−1.617	–
VEGF	1.617	1.562	−5.111
VEGF-D	1.849	–	–
XEDAR	8.613	–	−2.045

Table S3. Different expression patterns between the two phases in CD4+ T cells.

Expression	Viremic Phase CD4+	Aviremic phase CD4+
Unique		
ICAM-3	12.19	–
IL-21R	−15.04	–
LIF	9.12	–
TGF-α	31.91	–
Eotaxin-2	–	−5.71
Fractalkine	–	14.72
IGFBP-2	–	8.36
IGF-I SR	–	4.18
I-TAC	–	7.14
MIP-1 delta	–	−41.33
XEDAR	–	8.61
Reverse trend		
CCL-28	−2.46	2.09
EDA-A2	1.79	−1.80
FGF-7	1.84	−3.22
GRO	−1.58	1.59
IGFBP-3	−3.94	1.63
IGFBP-4	3.01	−2.25
IL-1 β	1.88	−2.11
IL-4	1.69	−7.65
Osteoprotegerin	−15.17	1.77
TECK	−8.94	2.00
TIMP-1	−3.04	1.95
TRAIL R4	−2.97	1.91
VEGF	−1.52	1.62
VEGF-D	−29.99	1.85
Same trend but Very High is one phase		
Dtk	−9.97	−4.92
Endoglin	−5.57	−1.74
FSH	2.08	6.72
MIP-1 α	1.87	3.19
MIP-1 β	1.63	3.23
PIGF	4.51	1.74
TGF- β 3	1.58	4.39

Table S4. Different expression patterns between the two phases in CD14+ T cells.

Expression	Viremic Phase CD14+	Aviremic Phase CD14+
Unique		
Dtk	37.76	
IGFBP-3	4.40	
MMP-13	10.33	
PIGF	−17.39	
SCF R	8.78	
TECK	−8.18	
TGF-α	8.39	
TRAIL R3	−3.90	
VEGF-D	−55.24	

Table S4. *Cont.*

Expression	Viremic Phase CD14+	Aviremic Phase CD14+
Unique		
Cathepsin S		−7.38
CCL14a		−5.04
Fractalkine		11.95
FSH		7.18
GCP-2		6.34
GM-CSF		4.72
Reverse trend		
EpCAM	6.00	−1.82
IGFBP-2	−2.51	1.86
MCP-4	−1.91	4.56
MIP-1 α	−2.08	1.58
MIP-1 delta	−3.61	1.51
MMP-7	36.80	−2.68
PDGF-BB	−1.88	2.64
Same trend but Very High is one phase		
ALCAM	−4.63	−2.59
ENA-78	−14.91	−2.82
Endoglin	−6.88	−2.17
GITR	−4.26	−1.75
GRO	−6.47	−1.82
IL-13 R α1	10.58	2.50
IL-17B	1.99	10.87
IP-10	4.17	3.46
I-TAC	9.88	8.26
NAP-2	1.62	3.71
TACE	−3.14	−9.88
TIMP-1	−6.60	−1.62
uPAR	−7.54	−1.62

Table S5. Different expression patterns between the two phases in CD8+ T cells.

Expression	Viremic CD8+	Aviremic CD8+
Unique		
Dtk	−15.49	–
EGF-R	7.87	–
FSH	4.20	–
MIP-1 delta	−5.58	–
ACE-2	–	5.19
BMP-4	–	−7.52
CCL14a	–	18.69
EDA-A2	–	−4.20
EGF	–	−33.68
FGF-7	–	20.01
FLRG	–	−9.49
Fractalkine	–	4.65
GCP-2	–	−4.30
GM-CSF	–	5.09
IGFBP-4	–	−7.52

Table S5. *Cont.*

Expression	Viremic CD8+	Aviremic CD8+
	Unique	
I-TAC	–	13.26
MCP-4	–	6.95
M-CSF	–	11.79
NT-4	–	−9.35
PARC	–	−19.78
PDGF-BB	–	−10.60
SCF	–	36.28
TACE	–	−86.26
TGF-β 3	–	−3.98
VEGF	–	−5.11
	Reverse trend	
ErbB2	−1.84	7.57
Fcr RIIB/C	−1.72	1.92
HGF	1.73	−1.67
IL-13 R α1	1.90	−2.79
Leptin R	2.43	−2.04
MIP-1 β	14.75	−2.67
MIP-3 α	−1.51	62.33
MMP-10	−4.11	3.02
XEDAR	1.98	−2.04
	Same trend but Very High is one phase	
CK β 8-1	7.25	5.64
IGFBP-2	1.57	7.91
IL-1 β	3.47	2.71
MIP-1 α	9.04	1.95
NAP-2	−1.73	−112.91

Table S6. Parallel comparison of differentially expressed proteins in CD4+ T cells, CD14+ monocytes, and CD8+ T cells during the viremic and aviremic phases.

ARRAY	Viremic Phase			Aviremic Phase		
Cell type	CD4 DE genes and % response	CD8 DE genes and % response	Monocytes DE genes and % response	CD4 DE genes and % response	CD8 DE genes and % response	Monocytes DE genes and % response
Protein array	39/62 (63%)	27/62 (43%)	40/62 (68%)	43/68 (63%)	41/68 (60%)	36/68 (53%)
